# Use of endoscopic ultrasound‐guided fine needle aspiration of the pancreas to diagnose a case of primary linitis plastica of the colon with retroperitoneal dissemination

**DOI:** 10.1002/deo2.12

**Published:** 2021-07-05

**Authors:** Masahiro Shitani, Jiro Ogino, Masakazu Akahonai, Mai Isosaka, Shigenori Ota, Yoshiko Tayama, Tomomi Ueki, Tetsuhiro Tsuruma, Takeya Adachi, Koichi Hirata, Hiroshi Nakase

**Affiliations:** ^1^ Department of Gastroenterology and Hepatology JR Sapporo Hospital Hokkaido Japan; ^2^ Department of Pathology JR Sapporo Hospital Hokkaido Japan; ^3^ Department of Surgery JR Sapporo Hospital Hokkaido Japan; ^4^ Department of Gastroenterology and Hepatology Sapporo Medical University School of Medicine Hokkaido Japan

**Keywords:** diffuse infiltrating type, scirrhous carcinoma, signet‐ring cell, pancreatic metastasis

## Abstract

A 54‐year‐old man had previously undergone curative sigmoidectomy for poorly differentiated adenocarcinoma with a signet‐ring cell component of the sigmoid colon, which was characterized morphologically by stenosis and inelasticity of the colon (linitis plastica). Six weeks after surgery, the patient developed stenosis of the right ureter. Disseminated sigmoid cancer was suspected, and chemotherapy was started. Nine months after initiation of chemotherapy, obstructive jaundice was observed which was due to stenosis of the distal bile duct (BD). Although computed tomography showed no evident metastatic lesion that could cause the stenosis, swelling of the entire pancreas was evident compared to that of 11 months earlier. Endoscopic ultrasound (EUS) also did not detect any focal masses in the head of the pancreas, although there was a diffuse hypoechoic change in the entire pancreas. Histopathology of the stenotic BD and biopsy specimen from the head of the pancreas showed no malignant cells. Two months after the initial endoscopic bile duct drainage, the patient was admitted again for epigastric pain. A second EUS fine needle aspiration (EUS‐FNA) of the head of the pancreas was performed and showed poorly differentiated carcinoma with some signet‐ring cells. This finding provided histological confirmation of a disseminated pancreatic lesion of the previously resected linitis plastica of the sigmoid colon. This is a rare case of disseminated pancreatic lesion from primary linitis plastica of the colon diagnosed by EUS‐FNA.

## INTRODUCTION

Linitis plastica is described as a scirrhous carcinoma with diffuse thickening and hardening of the bowel wall and characterized morphologically by stenosis and inelasticity of the colon. The incidence of linitis plastic is rare, with a reported prevalence rate of 0.1%–0.2%.^1^, ^2^ For pancreatic malignancies, only 1.8% are metastatic pancreatic tumors.^3^ Metastatic pancreatic lesions derived from tumors such as renal cell carcinoma and nonsmall cell lung cancer are known to have well‐defined margins,^4^ and it has been reported that histological evaluation of a biopsy specimen is useful for definitive diagnosis.^5^ However, there have been no reports of metastatic or disseminated pancreatic lesions from linitis plastica of the colon. In this paper, a case of a disseminated pancreatic lesion from primary linitis plastica of the sigmoid colon diagnosed by endoscopic ultrasound‐guided fine needle aspiration (EUS‐FNA) is reported, together with the imaging characteristics on computed tomography (CT), magnetic resonance imaging (MRI), and endoscopic ultrasound (EUS).

## CASE REPORT

**FIGURE 1:**
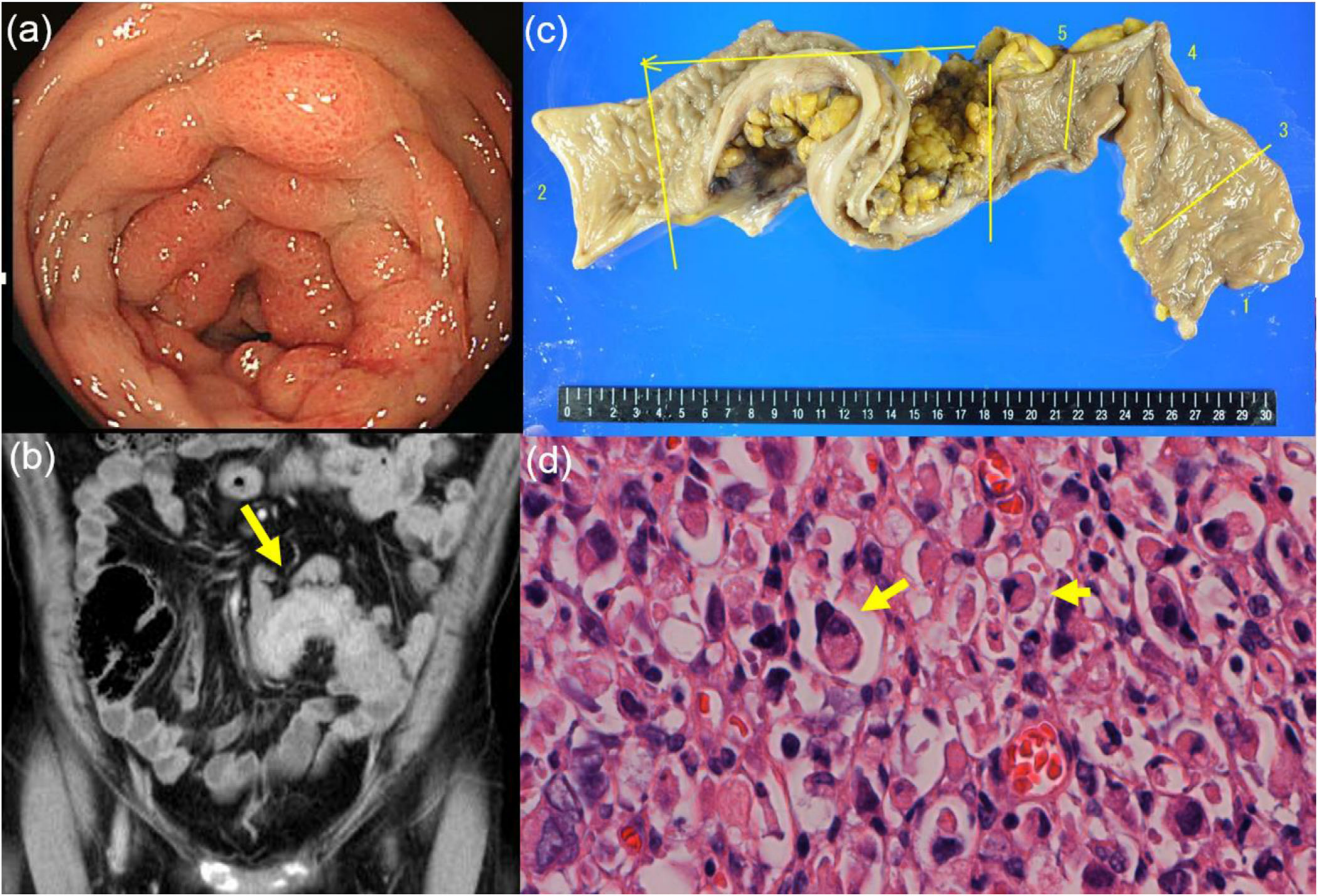
(a) Colonoscopy shows diffuse narrowing of the sigmoid colon lumen with hyperemia and a nodule‐like appearance. (b) Abdominal computed tomography (CT) shows long segmental thickening of the sigmoid colon (arrow). (c) Resected specimen of the sigmoid colon and (d) hematoxylin and eosin staining show poorly cohesive carcinoma and tumor‐cell infiltration to the submucosa; signet‐ring cells are seen (arrow)

A 54‐year‐old male visited our hospital complaining of constipation and small‐caliber stools for 1 month. Colonoscopic examination showed marked narrowing of the sigmoid colon lumen with edematous mucosa (Figure 1a). Histological examination of a biopsy specimen demonstrated poorly differentiated adenocarcinoma. Abdominal CT showed long segmental narrowing of the sigmoid colon (Figure 1b), no metastatic lesions in the lung and liver, and no ascites. The patient was diagnosed with primary linitis plastica of the sigmoid colon with poorly differentiated adenocarcinoma, and sigmoidectomy was performed (Figure 1c). Histopathological examination of the resected specimens showed poorly differentiated adenocarcinoma with signet‐ring cells (Figure 1d, arrow) infiltrating into the colonic mucosa with venous and lymph duct invasion and lymph node metastasis. The final diagnosis was pT3, pN3, cM0, stage 3b, according to the 8th edition of the TNM classification, with curative resection.

**FIGURE 2:**
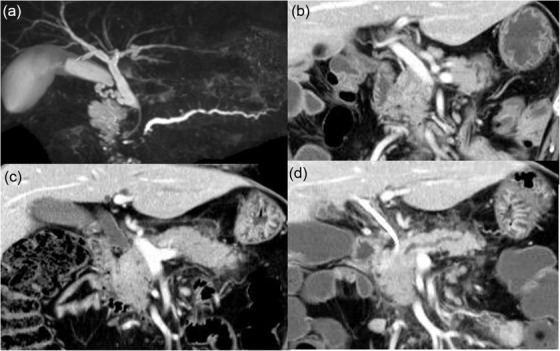
(a) Magnetic resonance cholangiopancreatography shows stenosis of the distal segments of the bile duct and main pancreatic duct and dilation of the upstream segments. (b) Coronal view of CT before the resection. (c) Coronal image of CT obtained 11 months after resection shows swelling of the entire pancreas. (d) CT image at the time of the second endoscopic ultrasound‐guided fine needle aspiration (EUS‐FNA) shows atrophic changes in the body and tail of the pancreas, peripancreatic soft tissue infiltration, and swelling of the head of the pancreas. *(a): After endoscopic biliary drainage. A plastic stent is placed in the common bile duct

**FIGURE 3:**
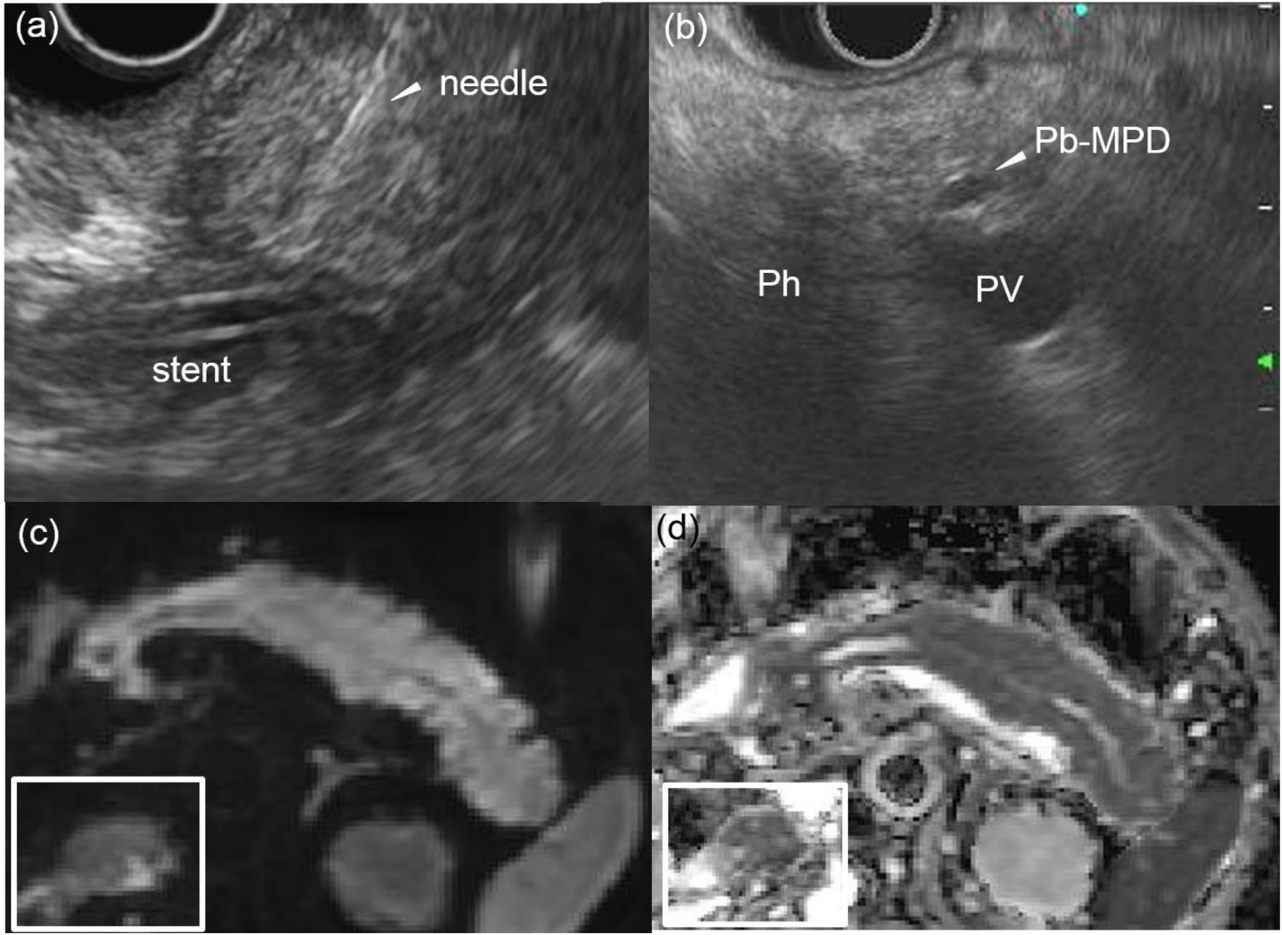
(a) Image obtained during the second EUS‐FNA from the descending duodenum. Head of the pancreas seemed to be enlarged and its outline changed to being rounder in shape compared to that of the first EUS. (b) EUS from the stomach shows hypoechoic parenchyma of the head of the pancreas, compared to the body of the pancreas. (c) Diffusion‐weighted images show moderate diffusion restriction in the body and tail of the pancreas, but not in the head of the pancreas (square). (d) Apparent diffusion coefficient values in the head of the pancreas (square) and other regions are not different. *Abbreviations: Ph, head of the pancreas; Pb‐MPD, main pancreatic duct at the body of the pancreas; PV, portal vein

**FIGURE 4:**
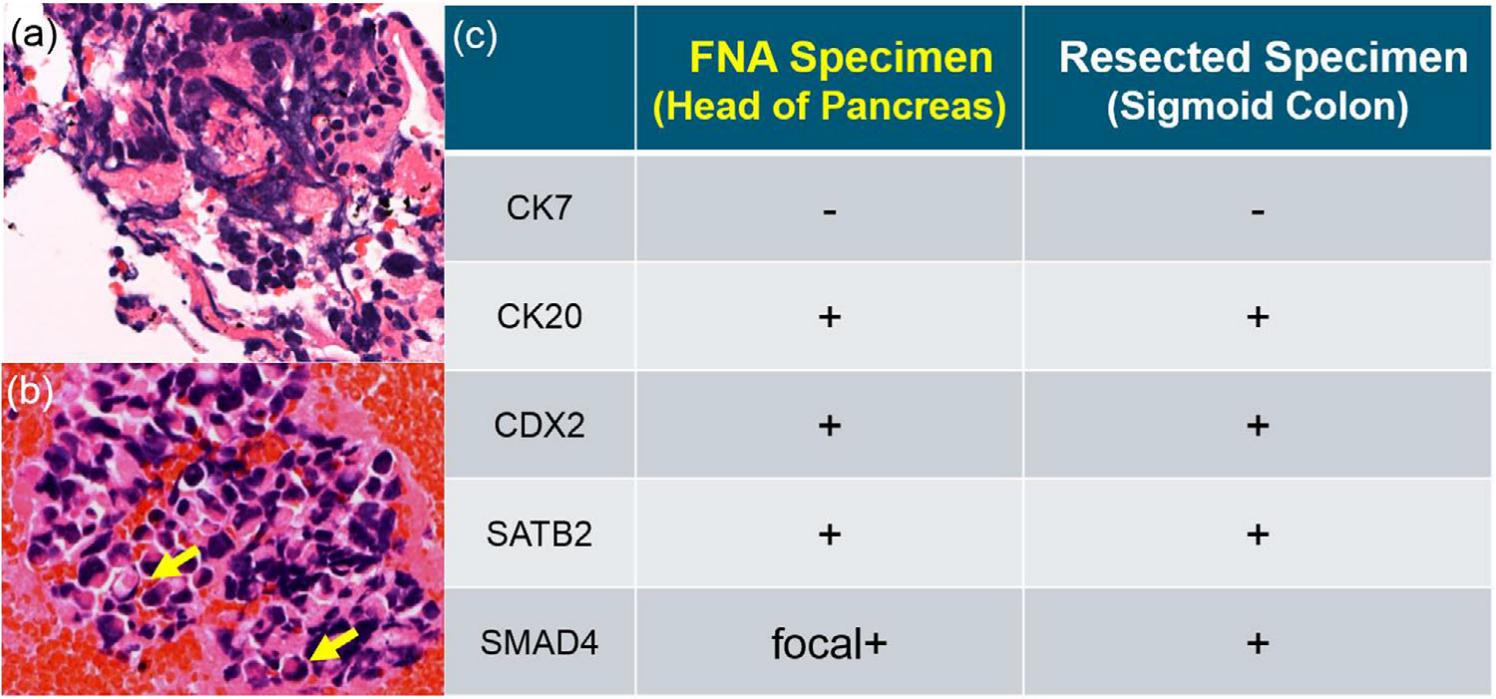
(a) Poorly differentiated carcinoma cells and (b) some signet‐ring cells (arrow) are seen on hematoxylin and eosin staining of biopsy specimens obtained by EUS‐FNA (200×). (c) Results of immunohistochemical analysis of the sigmoid colon and the pancreatic lesion

CT performed 6 weeks after surgery showed right renal pelvis dilation due to stenosis of the right ureter and para‐aortic and mesenteric lymphadenopathy, which suggested retroperitoneal dissemination. This prompted initiation of FOLFOX (5‐FU, leucovorin and oxaliplatin) + cetuximab regimen. After three courses of the said regimen, lymphadenopathy and urinary tract stenosis markedly improved. During the course of chemotherapy, no obvious recurrence was observed, and the tumor markers remained normal for 9 months. Eleven months after surgery, lymphadenopathy of the right inguinal lymph nodes and obstructive jaundice were noted. Magnetic resonance cholangiopancreatography showed dilation of the bile duct (BD) and the main pancreatic duct (MPD), suggesting a lesion in the head of the pancreas (Figure 2a). Although CT did not show any mass in the head of the pancreas that could cause obstruction, it demonstrated swelling of the entire pancreas (Figure 2c, Figure S1c and d) compared to that of 11 months earlier (Figure 2b, Figure S1a and b). EUS (GF‐UCT260; Olympus, Tokyo, Japan) also showed no distinct masses, but the entire pancreatic parenchyma was hypoechoic (Figure S2). Compared to the head of the pancreas (Figure 3c, square), diffusion‐weighted MRI demonstrated slight abnormal diffusion restriction in the body and tail of the pancreas (Figure 3c), but no areas showed focal attenuation of the apparent diffusion coefficient (ADC) value in the pancreas (Figure 3d). Histological examination of a BD biopsy performed during endoscopic bile duct drainage (EBD) and cytopathological examination of EUS‐FNA found no malignant cells. There were also no malignant cells in a right inguinal lymph node that was excised. Two months after the EBD, the patient was admitted with epigastric pain. CT showed swelling of the adrenal glands and peripancreatic soft tissue infiltration (Figure 2d). In contrast to the atrophic change of the body and tail of the pancreas, swelling and mass formation were evident in the head of the pancreas (Figure S1e and f). A second EUS‐FNA of the head of the pancreas was performed using an Olympus 22‐G FNA needle (NA‐U200H; Olympus, Tokyo, Japan). The results revealed that the head of the pancreas seemed to be enlarged and its outline changed to being rounder in shape (Figure 3a and b and Supporting Information Video) compared to that of the first EUS. The biopsy specimens showed poorly differentiated carcinoma and some signet‐ring cells (Figure 4a and b). Tumor cells were negative for cytokeratin seven and positive for *cytokeratin 20*, *CDX2*, *SATB2*, and *SMAD4* (Figure 4c, Figure S3), suggesting that the tumor cells originated from the colorectal epithelium. Immunohistochemical evaluation of the biopsied specimen was consistent with the histology of the previously resected linitis plastica of the sigmoid colon. The patient's liver function gradually deteriorated due to multiple stenoses of intrahepatic bile ducts, which appeared within 3 weeks after his second hospitalization. The patient eventually died of liver failure 15 months after initial surgical resection.

## DISCUSSION

Laufman et al first reported primary linitis plastica of the colon in 1951.^6^ It was described as a scirrhous carcinoma with diffuse thickening and hardening of the bowel wall. The histological characteristics included a scirrhous reaction by poorly differentiated adenocarcinoma to signet‐ring cell carcinoma.^2^ It is known to develop at an earlier age and metastasize rapidly to lymph nodes and disseminate to the peritoneum.

The reported incidence of patients with colorectal cancer exhibiting a component of signet‐ring cells comprising <50% of the tumor mass is 5%.^7^ This histological type is known to have poor survival rates compared to common histological types consisting of well‐differentiated and moderately differentiated adenocarcinoma cells. The majority of metastatic pancreatic lesions originated from renal cell carcinomas (63.6%, 14/22), followed by colon adenocarcinomas (9.0%, 2/22) and nonsmall cell lung carcinomas (4.5%, 1/22).^8^ Sakai et al reviewed a series of 59 Japanese cases of pancreatic metastasis from colorectal cancer and reported that in 91.5% (54/59) of the cases, the histology was well‐differentiated and moderately differentiated adenocarcinoma, and the CT imaging showed a lobular mass with poor enhancement,^9^ different from the present case. Prior to this, there have been no previous reports of pancreatic metastases or dissemination of primary linitis plastica of the colon.

The usefulness and high accuracy of EUS‐FNA in diagnosing the etiology of several diseases have been widely accepted,^5^ and it can be used to investigate the etiology of pancreatic lesions. In the present case, biopsy specimens from the head of the pancreas obtained by the first EUS‐FNA showed normal acinar cells, whereas those by the second EUS‐FNA showed poorly differentiated carcinoma with signet‐ring cells. It is suspected that the reason for the success of the second EUS‐FNA in arriving at a diagnosis is an increase in the number of tumor cells in the puncture site. Drastic changes in CT findings and rapid disease progression after the second EUS‐FNA may reflect the aggressive cell proliferation in the retroperitoneal space. Therefore, further examination of similar cases may be needed to establish the number of biopsies and the required puncture procedures to accurately diagnose metastatic lesions with scirrhous carcinoma.

The characteristics of pancreatic metastases are reported to be lesions with regular borders, lack of retention cysts, and presence of a nondilated MPD.^4^ In the present case, despite the MPD and BD that were markedly stenosed in the head of the pancreas, EUS neither showed any well‐demarcated lesion in the area nor any masses with poor enhancement on CT. These features are rather reminiscent of so‐called mass‐forming pancreatitis (MFP). Similar to MFP, there was no focal attenuation of ADC values throughout the pancreas, including the head of the pancreas.^10^ These imaging characteristics could suggest the characteristics of disseminated pancreatic lesions of linitis plastica of the colon.

In summary, this is the first report of a case of disseminated pancreatic lesions of linitis plastica of the colon diagnosed by EUS‐FNA, with characteristic features on CT, MRI, and EUS. Repeated EUS‐FNA might be necessary to make the histological diagnosis because the poorly cohesive cancer cells infiltrate diffusely and do not form masses in the metastatic organs.

## CONFLICT OF INTEREST

The authors declare no conflict of interest.

## FUNDING INFORMATION

None.

## Supporting information




**Supplemental Video**. Second endoscopic ultrasound‐guided fine needle aspiration of the head of the pancreas is performed from the descending duodenum.

**Supplemental Fig. 1**. Axial images of computed tomography demonstrating the body and tail of the pancreas (upper) and head of the pancreas (bottom) obtained before resection (a, b), 11 months after resection (c, d), and at the time of the second Endoscopic ultrasound‐guided fine needle aspiration (e, f). (e) Soft tissue infiltration in the retroperitoneal space (arrowhead) and the swelling of the adrenal gland (arrow) are seen. (f) Enlargement of the head of the pancreas (arrowhead) is evident.
**Supplemental Fig. 2**. Endoscopic ultrasound (EUS) images at the first EUS fine needle aspiration. (a) EUS from the descending duodenum shows hypoechoic parenchyma and no well‐demarcated lesion in the head of the pancreas where the main pancreatic duct (MPD) is constricted. (b) EUS from the stomach shows a dilated MPD and hypoechoic parenchyma in the body of the pancreas. *Abbreviations: Ph‐MPD, main pancreatic duct at the head of the pancreas; Pb‐MPD, main pancreatic duct at the body of the pancreas
**Supplemental Fig. 3**. Results of immunohistochemical analysis of the sigmoid colon and the pancreatic lesion. (a‐d) Biopsy specimens obtained by the endoscopic ultrasound‐guided fine needle aspiration (x100). (e‐h) Previously resected specimen (x100)
