# Supplementary figures and images for: Use of endoscopic ultrasound‐guided fine needle aspiration of the pancreas to diagnose a case of primary linitis plastica of the colon with retroperitoneal dissemination
Source: DEN Open. 2021 Jul 5;2(1):e12. doi: 10.1002/deo2.12 (PMC8828226; doi:10.1002/deo2.12)

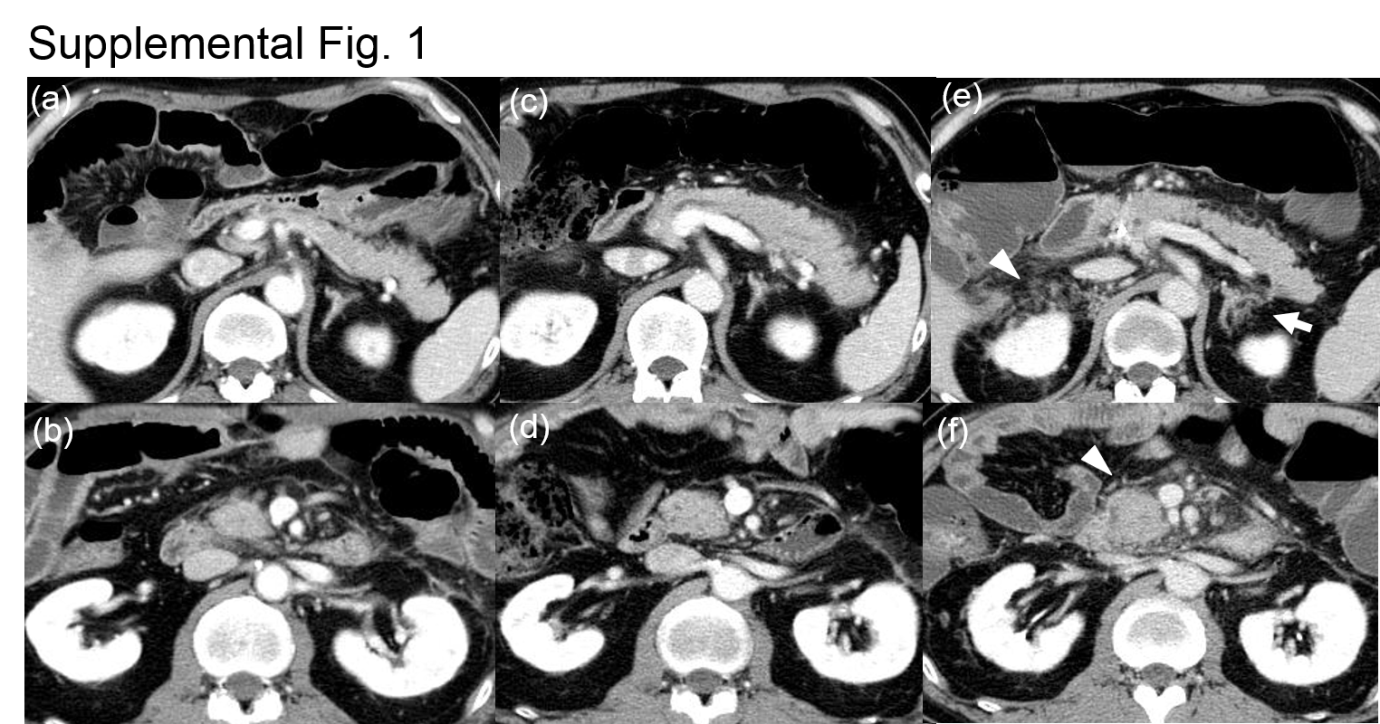


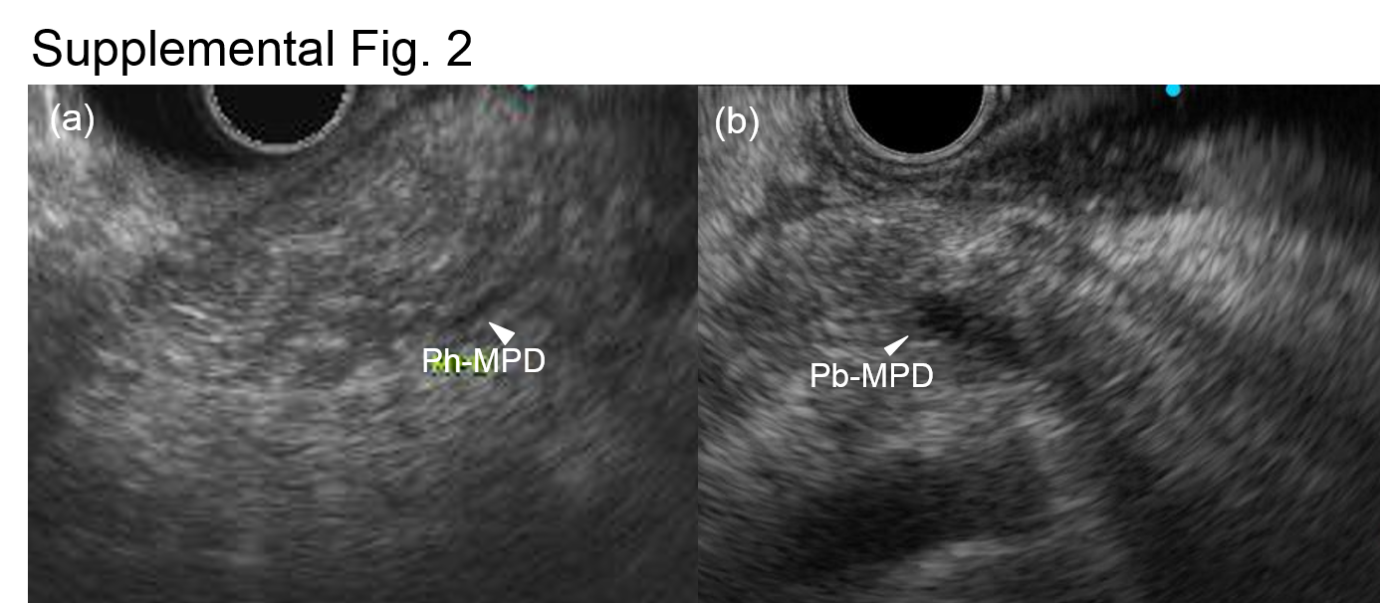


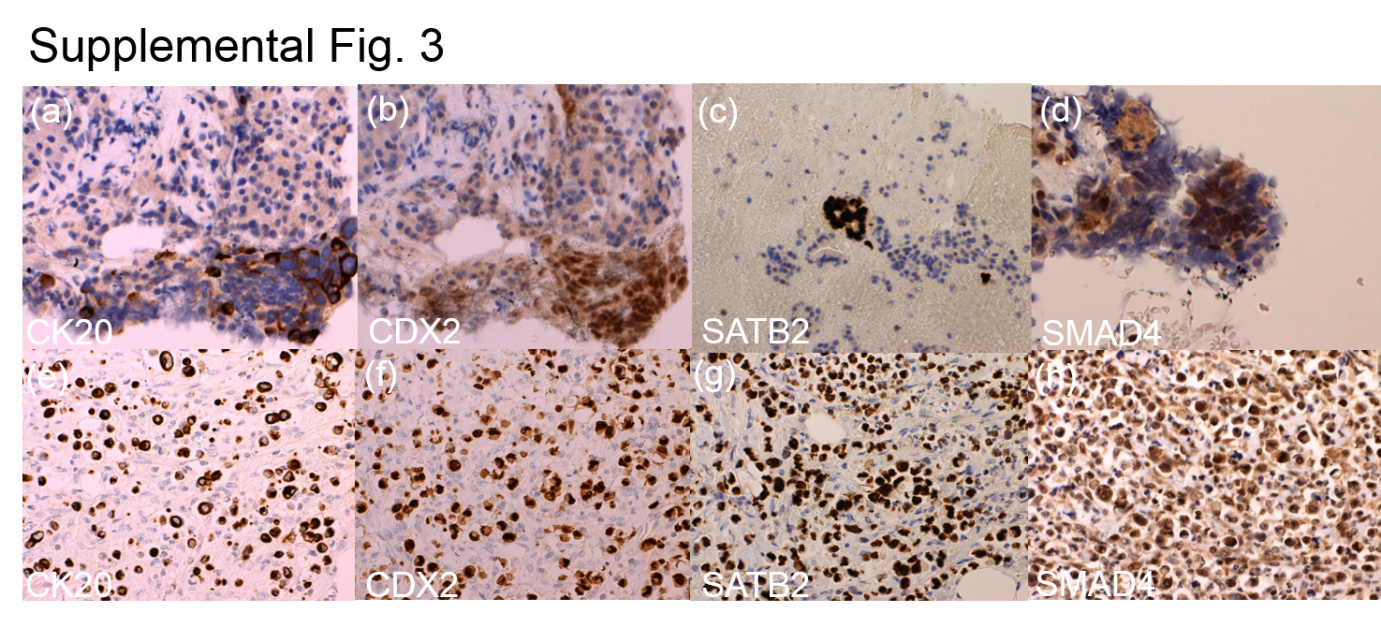

Supplement: Supplementary file 2 — Supplemental Fig. 1. Axial images of computed tomography demonstrating the body and tail of the pancreas (upper) and head of the pancreas (bottom) obtained before resection (a, b), 11 months after resection (c, d), and at the time of the second Endoscopic ultrasound‐guided fine needle aspiration (e, f). (e) Soft tissue infiltration in the retroperitoneal space (arrowhead) and the swelling of the adrenal gland (arrow) are seen. (f) Enlargement of the head of the pancreas (arrowhead) is evident. Supplemental Fig. 2. Endoscopic ultrasound (EUS) images at the first EUS fine needle aspiration. (a) EUS from the descending duodenum shows hypoechoic parenchyma and no well‐demarcated lesion in the head of the pancreas where the main pancreatic duct (MPD) is constricted. (b) EUS from the stomach shows a dilated MPD and hypoechoic parenchyma in the body of the pancreas. *Abbreviations: Ph‐MPD, main pancreatic duct at the head of the pancreas; Pb‐MPD, main pancreatic duct at the body of the pancreas Supplemental Fig. 3. Results of immunohistochemical analysis of the sigmoid colon and the pancreatic lesion. (a‐d) Biopsy specimens obtained by the endoscopic ultrasound‐guided fine needle aspiration (x100). (e‐h) Previously resected specimen (x100) [file DEO2-2-e12-s001.docx]
